# Supplementary material for: Liver protects neuron viability and electrocortical activity in post-cardiac arrest brain injury
Source: EMBO Mol Med. 2024 Sep 19;16(10):2322–48. doi: 10.1038/s44321-024-00140-z (PMC11479250; doi:10.1038/s44321-024-00140-z)
Supplement: Supplementary file 1 — Appendix [file 44321_2024_140_MOESM1_ESM.pdf]

Appendix information for

‘*Liver protects neuron viability and electrocortical activity in post-cardiac arrest brain injury*’

This PDF file includes:

Appendix Figures S1 to 6

Appendix Tables S1 to 3

Contents

Appendix Figure S1. 1
Appendix Figure S2. 2
Appendix Figure S3. 4
Appendix Figure S4. 5
Appendix Figure S5. 6
Appendix Figure S6. 7
Appendix Table S1 8
Appendix Table S2 8
Appendix Table S3 9

Appendix figures and figure legends

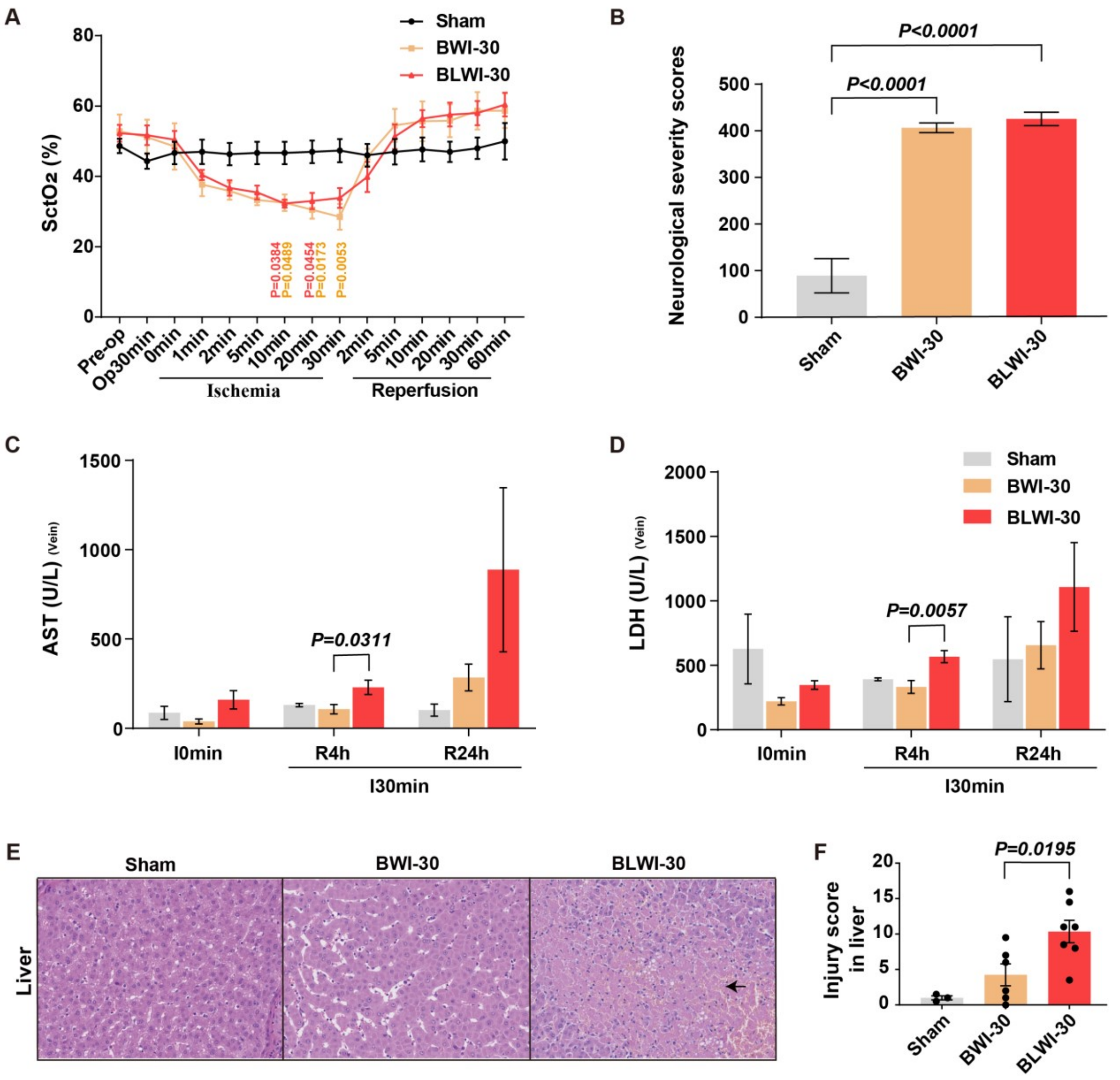

Appendix Figure S1. Assessment of global cerebral ischemia *in vivo* using models with or without liver ischemia.

(A) Cerebral tissue oxygen saturation ($SctO_2$ (%)) of the *in vivo* model. Sham, no ischemia, *n* = 3; BWI-30, brain with 30-minutes warm ischemia, *n* = 6; BLWI-30, brain and liver with 30-minutes warm ischemia, *n* = 7; Op, operation; Mean ± SEM, 2-way ANOVA analysis; versus Sham. **(B)** Neurological severity scores were assessed by two individuals at 6 hours after reperfusion following 30 minutes of ischemia. Sham, *n* = 3; BWI-30, *n* = 6; BLWI-30, *n* = 5. **(C, D)** Serum aminotransferase (AST) (**C**) and lactate dehydrogenase (LDH) **(D)** levels. I, ischemia; R, reperfusion. **(E)** Haematoxylin and eosin staining of the liver. The arrowhead

pointed to the damaged tissue. **(F)** The injury scores for the mean of two fields in the liver. **(B) to (D), and (F)**, Mean ± SEM, two-tailed ratio unpaired t-test. **(C), (D), and (F)**, Sham, *n* = 3; BWI-30, *n* = 6; BLWI-30, *n* = 7. All replicates shown were biological replicates.

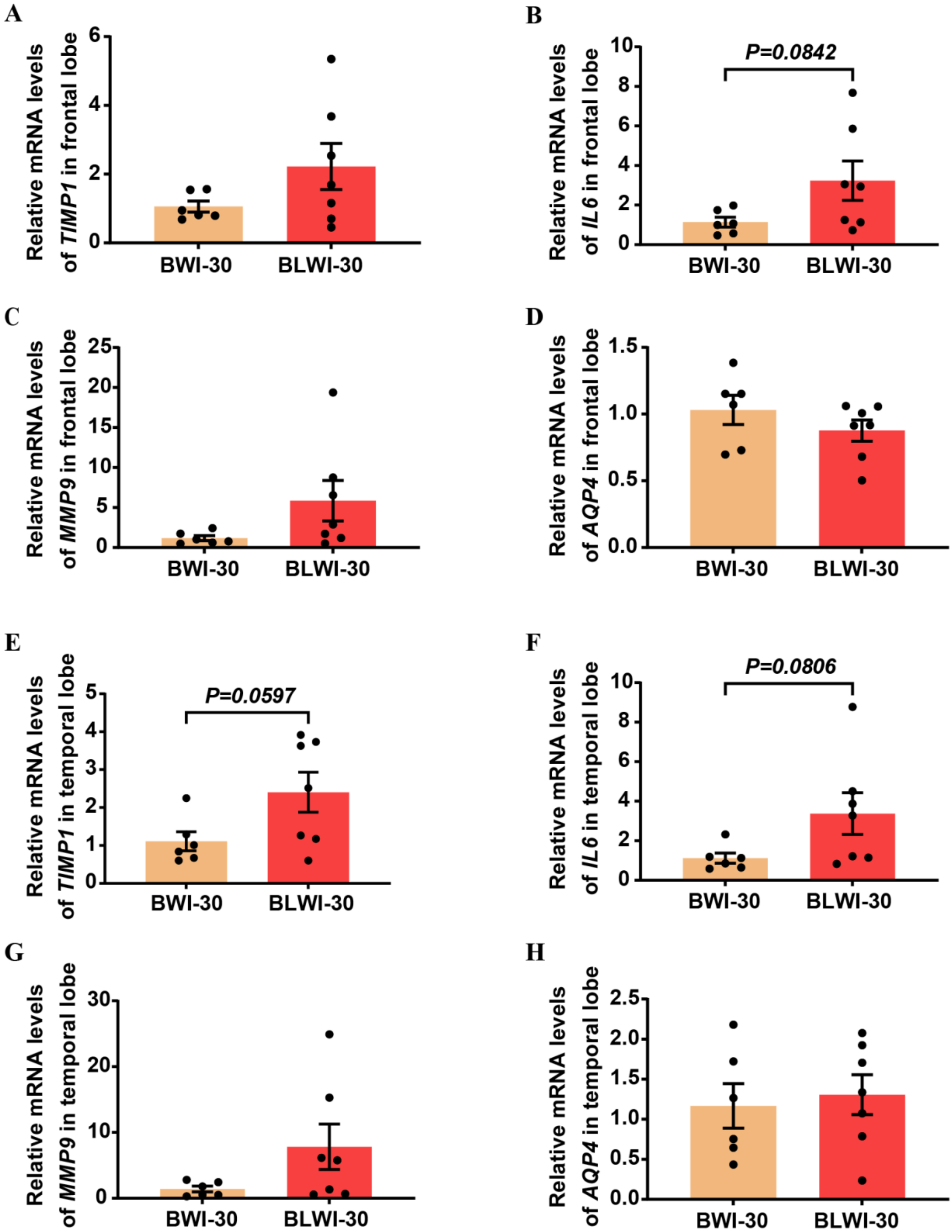

Appendix Figure S2. Expression of genes related to blood-brain barrier (BBB) damage in the pig cerebral cortex.

(A-D) Relative mRNA levels of *TIMP1*, *IL6*, *MMP9*, and *AQP4* in the frontal lobe. **(E-H)** Relative mRNA levels of *TMP1*, *IL6*, *MMP9*, and *AQP4* in the temporal lobe. **(A-H)** Mean ± SEM, two-tailed ratio unpaired *t*-test; BWI-30, brain with 30-minutes warm ischemia, n = 6; BLWI-30, BLWI-30, brain and liver with 30-minutes warm ischemia, n = 7. All replicates shown were biological replicates.

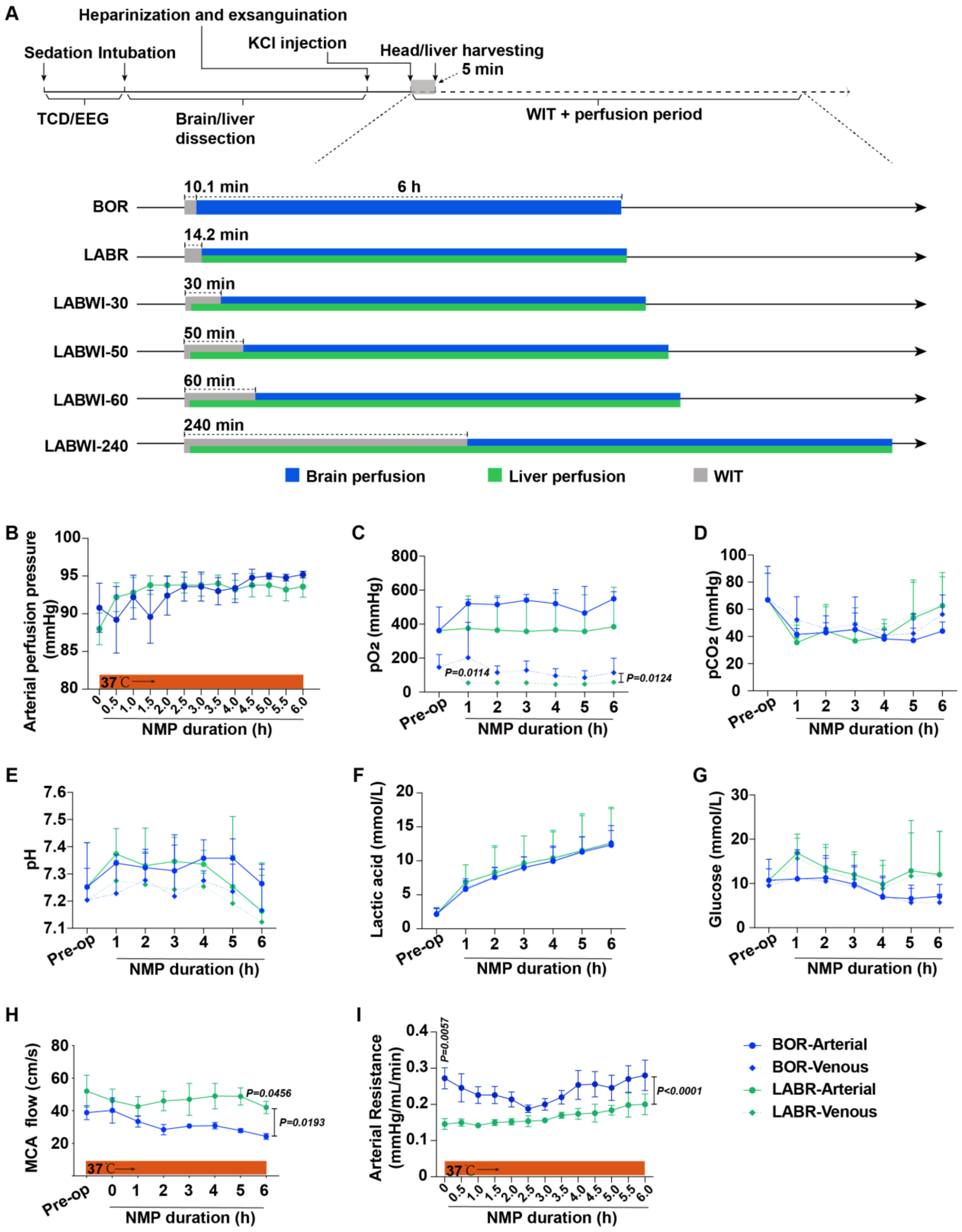

Appendix Figure S3. ***Ex vivo*** **NMP of pig brains can restore vascular circulation and metabolic activity.**

(A) Schematic depicting the experimental workflow and conditions. WIT, warm ischemia time; BOR, brain-only controls with rapid NMP; LABR, liver-assisted brains with rapid NMP;

LABWI-30/50/60/240, liver-assisted brain groups in which NMP was preceded by 30-240 minutes WIT; EEG, electroencephalography; TCD, transcranial Doppler. **(B)** Pressure in the common carotid artery trunk. **(C-G)** Paired measurements of BOR and LABR in arterial or venous samples indicating the arteriovenous gradients of pO_2 (**C**), pCO_2 (**D**), pH (**E**), lactic acid (**F**) and glucose (**G**) in the *ex vivo* brain NMP system. (**H**) The middle cerebral artery (MCA) of the five brains during *ex vivo* NMP. (**I**) Vascular resistance of the common carotid artery trunk. **(B) to (I)**, n = 5 biological replicates; Mean ± SEM, 2-way ANOVA analysis; BOR versus LABR.

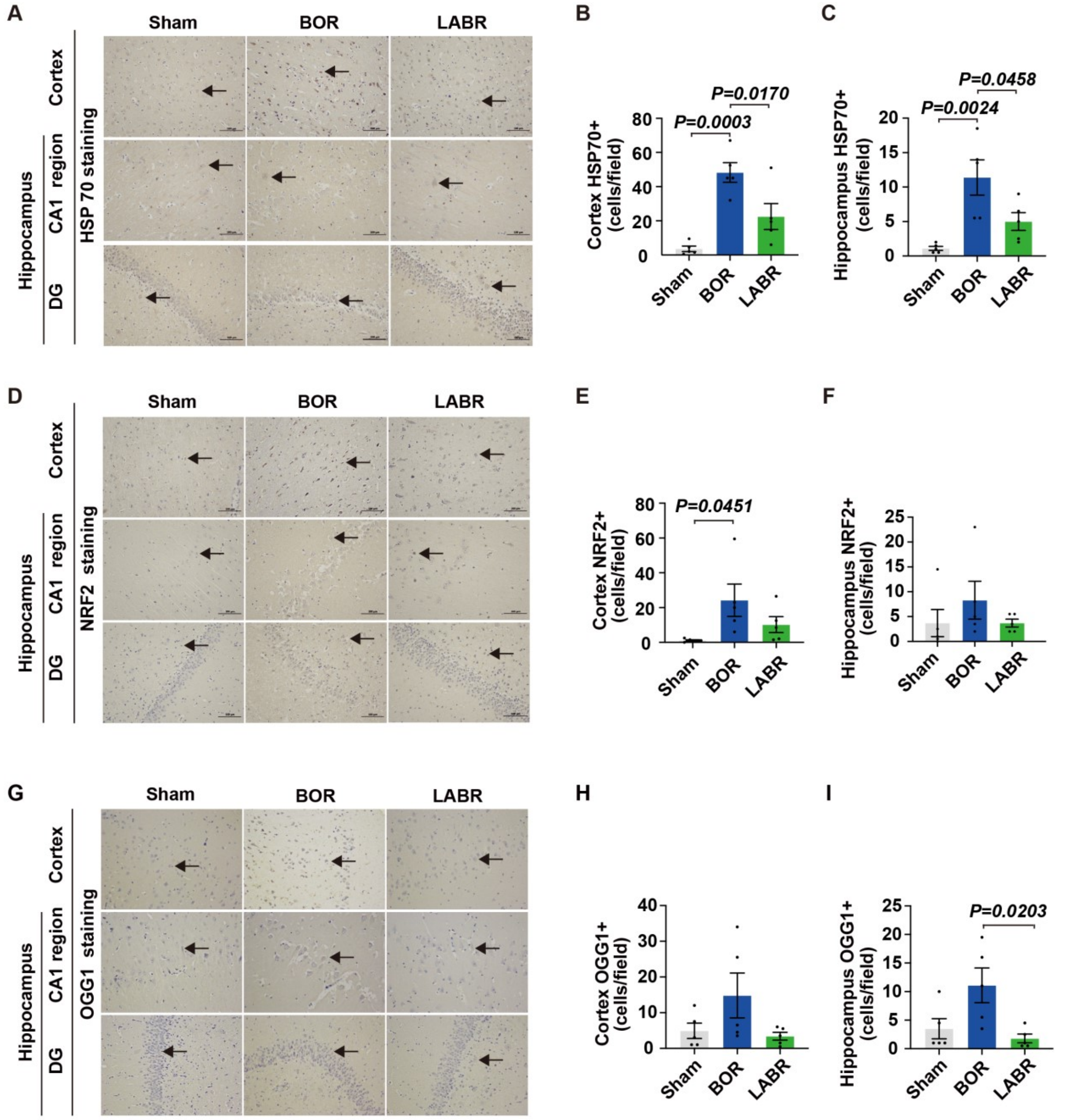

Appendix Figure S4. Staining for hypoxia-induced markers in perfused pig brains.

(A-C) Immunohistochemistry analysis of HSP70 indicated the degree of heat shock stimulation. The upregulation of HSP70 was shown as deep brown staining. The number of cells with upregulated expression of HSP70 in the cortex (**B**) and hippocampus (**C**). **(D-F)** Immunohistochemistry analysis of nuclear factor erythroid 2 like 2 (NRF2) demonstrates the degree of oxidative stress. NRF2 was shown as brown staining. The number of cells with upregulated expression of NRF2 in the cortex (**E**) and hippocampus (**F**). **(G-I)** Immunohistochemistry analysis of DNA repairing 8-oxoguanine DNA glycosylase (OGG) demonstrates the repair of oxidative damage. OGG1 was shown as brown staining. The number of cells showing upregulated expression of OGG1 in the cortex (**H**) and hippocampus (**I**). **(A-I)** Two fields (200×) of each brain were used for counting. $n = 5$ biological replicates; Mean ± SEM, two-tailed ratio unpaired t-test; *P<0.05, **P<0.01, ***P<0.001.

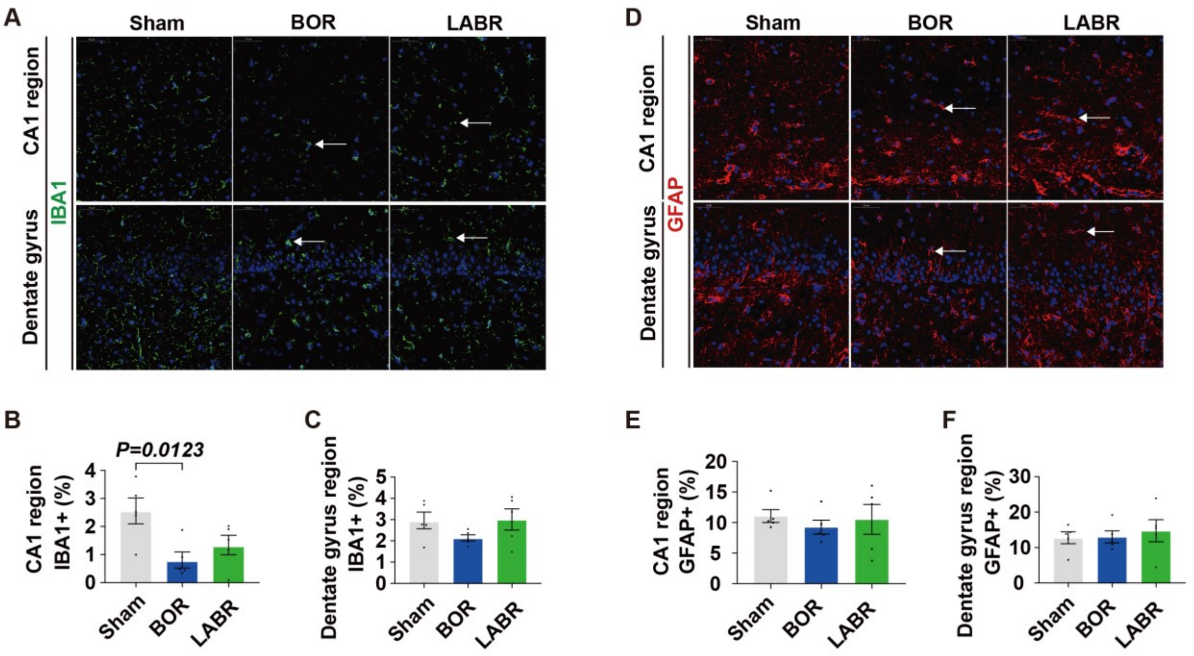

Appendix Figure S5. Viability of microglia and astrocytes.

(A-C) Immunofluorescence staining for IBA1 in the hippocampus of pigs. IBA1-positive cells were counted in the CA1 field (**B**) and dentate gyrus (**C**). **(D-F)** Immunofluorescence staining for GFAP in the hippocampus of pigs. The fluorescence area of GFAP was measured in the CA1

field (**E**) and dentate gyrus (**F**). Two fields of view (200× magnification) from each brain were used for cell counting. $n = 5$ biological replicates; Mean ± SEM, two-tailed ratio unpaired t-test.

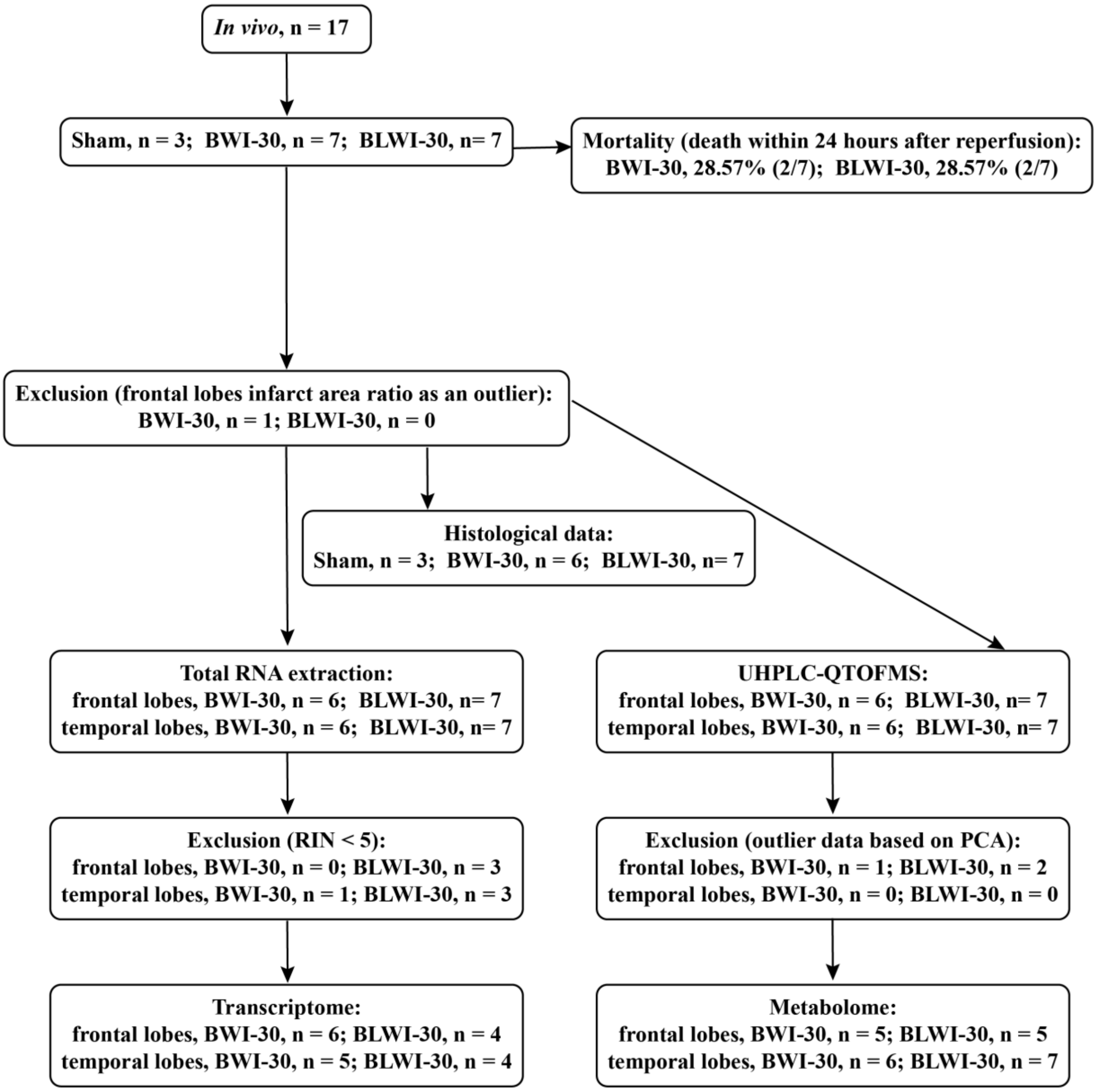

Appendix Figure S6. The number of pigs and data exclusion for specific analyses.
BWI-30, 30-min brain warm ischemia; BLWI-30, 30-min brain and liver warm ischemia; RIN, RNA integrity number; UHPLC-QTOFMS, ultra-high-performance liquid chromatography quadrupole time-of-flight mass spectrometry; PCA, principal component analysis.

Appendix tables

Appendix Table S1. Primer sequences of the genes**.**

Gene Name	Primer sequences	
Aqp4	Forward	CCGGCGGCCTTTATGAGTAT
Aqp4	Reverse	TTCTGTTGTCATCCGCCTCC
Gapdh	Forward	GTCGGAGTGAACGGATTTGGC
Gapdh	Reverse	CTTGCCGTGGGTGGAATCAT
Il6	Forward	CTGCAGTCACAGAACGAGTG
Il6	Reverse	CGGCATCAATCTCAGGTGCC
Mmp9	Forward	ACTTCGGAAACGCAAAAGGC
Mmp9	Reverse	AAGAGTCTCTCGCTAGGGCA
Timp1	Forward	TCACTGCTTGTGGACAGACC
Timp1	Reverse	GAACACTGTGCAGGTTTCGG

Appendix Table S2. Composition of the perfusate related to **Fig. 3A.**

Component	Volume/dose
Blood	800–1000 mL
Gelofusine	500 mL
Calcium chloride	1.5 g
Magnesium sulphate	0.75 g
5% Bicarbonate	40–50 mL
Compound amino acid	200 mL
Heparin	37500 U
Metronidazole	0.5 g
Methylprednisolone	500 mg
Sulperazone	1.5 g

Appendix Table S3. Electroencephalogram (EEG) scores were assessed at Pre-operation (Pre-op) and at 0.5, 1, 2, 3, 4, 5 and 6 hours (h) after normothermic machine perfusion (NMP) for all pigs. The statistical analysis of EEGs is presented in **Fig. 4F and Fig. EV2**, while EEG scores are detailed in **Fig. 4G**. No brainwaves = 0 points; δ wave = 1 point; θ wave = 2 points; α wave = 3 points; β wave = 4 points.

Group	Pig	NMP Duration	Brain Wave	EEG Score
BOR group	Pig 1	Pre-op	α, β	7
		30 min	α, β	7
		1 h	α, β	7
		2 h	θ, α	5
		3 h	α, β	7
		4 h	δ, θ	3
		5 h	δ, θ	3
		6 h	δ	1
	Pig 2	Pre-op	α, β	7
		30 min	α, β	7
		1 h	α, β	7
		2 h	θ, α	5
		3 h	α, β	7
		4 h	δ, θ	3
		5 h	θ	2
		6 h	δ	1
	Pig 3	Pre-op	α, β	7
		30 min	α, β	7
		1 h	δ, θ	3
		2 h	δ, θ	3
		3 h	α, β	7
		4 h	α, β	7
		5 h	θ, α	5

		6 h	δ	1
	Pig 4	Pre-op	α, β	7
		30 min	α, β	7
		1 h	α, β	7
		2 h	θ, α	5
		3 h	θ	2
		4 h	δ, θ	3
		5 h	δ	1
		6 h	δ	1
	Pig 5	Pre-op	α, β	7
		30 min	α, β	7
		1 h	α, β	7
		2 h	θ, α	5
		3 h	α, β	7
		4 h	α, β	7
		5 h	θ, α	5
		6 h	θ, α	5
LABR group	Pig 1	Pre-op	α, β	7
		30 min	δ, θ	3
		1 h	θ, α	5
		2 h	θ, α	5
		3 h	θ, α	5
		4 h	α, β	7
		5 h	α, β	7
		6 h	α, β	7
	Pig 2	Pre-op	α, β	7
		30 min	θ, α	5
		1 h	α, β	7
		2 h	θ, α	5

		3 h	θ, α	5
		4 h	θ, α	5
		5 h	α, β	7
		6 h	α, β	7
	Pig 3	Pre-op	α, β	7
		30 min	δ, θ	3
		1 h	α, β	7
		2 h	α, β	7
		3 h	θ, α	5
		4 h	θ, α	5
		5 h	α, β	7
		6 h	α, β	7
	Pig 4	Pre-op	α, β	7
		30 min	δ, θ	3
		1 h	α, β	7
		2 h	θ, α	5
		3 h	α, β	7
		4 h	α, β	7
		5 h	θ, α	5
		6 h	θ, α	5
	Pig 5	Pre-op	θ, α	5
		30 min	δ, θ	3
		1 h	α, β	7
		2 h	α, β	7
		3 h	α, β	7
		4 h	α, β	7
		5 h	α, β	7
		6 h	α, β	7
LABWI-30	Pig 1	Pre-op	α, β	7

group		30 min	θ, α	5
		1 h	θ, α	5
		2 h	α, β	7
		3 h	α, β	7
		4 h	α, β	7
		5 h	θ, α	5
		6 h	α, β	7
	Pig 2	Pre-op	θ, α	5
		30 min	θ, α	5
		1 h	θ, α	5
		2 h	δ, θ	3
		3 h	θ, α	5
		4 h	θ, α	5
		5 h	α, β	7
		6 h	α, β	7
	Pig 3	Pre-op	α, β	7
		30 min	α, β	7
		1 h	θ, α	5
		2 h	θ, α	5
		3 h	α, β	7
		4 h	δ, θ	3
		5 h	θ, α	5
		6 h	α, β	7
	Pig 4	Pre-op	α, β	7
		30 min	δ, θ	3
		1 h	θ, α	5
		2 h	δ, θ	3
		3 h	δ, θ	3
		4 h	θ, α	5

		5 h	θ, α	5
		6 h	δ, θ	3
	Pig 5	Pre-op	θ, α	5
		30 min	δ, θ	3
		1 h	δ, θ	3
		2 h	θ, α	5
		3 h	α, β	7
		4 h	α, β	7
		5 h	α, β	7
		6 h	α, β	7
LABWI-50 group	Pig 1	Pre-op	α, β	7
		30 min	θ, α	5
		1 h	θ, α	5
		2 h	α, β	7
		3 h	α, β	7
		4 h	α, β	7
		5 h	α, β	7
		6 h	α, β	7
	Pig 2	Pre-op	θ, α	5
		30 min	δ, θ	3
		1 h	δ, θ	3
		2 h	α, β	7
		3 h	α, β	7
		4 h	θ, α	5
		5 h	θ, α	5
		6 h	α, β	7
	Pig 3	Pre-op	α, β	7
		30 min	δ, θ	3
		1 h	δ, θ	3

		2 h	δ, θ	3
		3 h	δ, θ	3
		4 h	δ, θ	3
		5 h	θ, α	5
		6 h	θ, α	5
	Pig 4	Pre-op	θ, α	5
		30 min	θ, α	5
		1 h	θ, α	5
		2 h	δ, θ	3
		3 h	θ, α	5
		4 h	θ, α	5
		5 h	θ, α	5
		6 h	θ, α	5
	Pig 5	Pre-op	θ, α	5
		30 min	δ, θ	3
		1 h	δ, θ	3
		2 h	θ, α	5
		3 h	α, β	7
		4 h	θ, α	5
		5 h	θ, α	5
		6 h	α, β	7
LABWI-60 group	Pig 1	Pre-op	α, β	7
		30 min	no	0
		1 h	δ, θ	3
		2 h	δ, θ	3
		3 h	δ, θ	3
		4 h	θ, α	5
		5 h	δ, θ	3
		6 h	δ	1

	Pig 2	Pre-op	α, β	7
		30 min	no	0
		1 h	δ, θ	3
		2 h	δ, θ	3
		3 h	θ, α	5
		4 h	θ, α	5
		5 h	δ, θ	3
		6 h	δ, θ	3
	Pig 3	Pre-op	θ, α	5
		30 min	no	0
		1 h	δ, θ	3
		2 h	δ, θ	3
		3 h	θ, α	5
		4 h	δ, θ	3
		5 h	δ, θ	3
		6 h	δ, θ	3
	Pig 4	Pre-op	α, β	7
		30 min	no	0
		1 h	δ, θ	3
		2 h	δ, θ	3
		3 h	θ, α	5
		4 h	δ, θ	3
		5 h	δ, θ	3
		6 h	δ	1
LABWI-240 group	Pig 1	Pre-op	α, β	7
		30 min	no	0
		1 h	no	0
		2 h	no	0
		3 h	no	0

	4 h	no	0
	5 h	no	0
	6 h	no	0
Pig 2	Pre-op	α, β	7
	30 min	no	0
	1 h	δ, θ	3
	2 h	δ, θ	3
	3 h	δ, θ	3
	4 h	δ	1
	5 h	δ, θ	3
	6 h	δ	1
Pig 3	Pre-op	α, β	7
	30 min	no	0
	1 h	δ	1
	2 h	δ, θ	3
	3 h	δ, θ	3
	4 h	δ, θ	3
	5 h	δ	1
	6 h	no	0
