# Supplementary material for: Liver protects neuron viability and electrocortical activity in post-cardiac arrest brain injury
Source: EMBO Mol Med. 2024 Sep 19;16(10):2322–48. doi: 10.1038/s44321-024-00140-z (PMC11479250; doi:10.1038/s44321-024-00140-z)
Supplement: Supplementary file 11 — Expanded View Figures [file 44321_2024_140_MOESM11_ESM.pdf]

Expanded View Figures

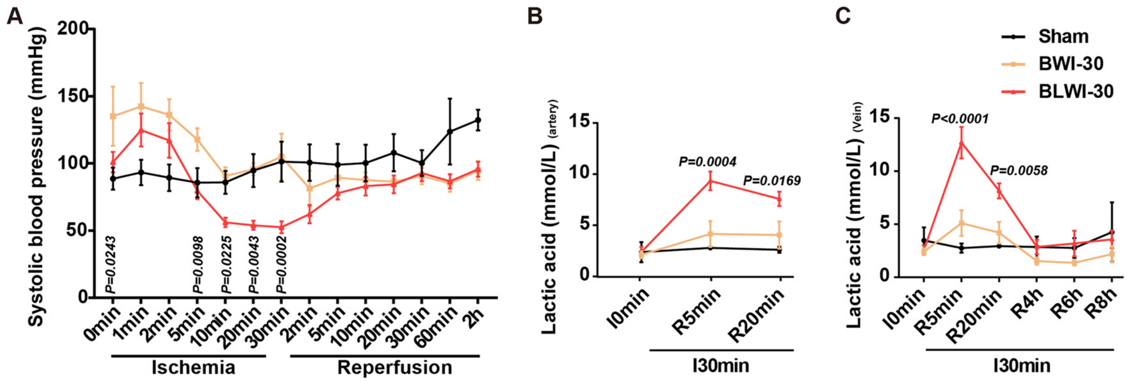

Figure EV1. Systolic blood pressure and lactic acid levels in the global cerebral ischemia model.

(**A**) Systolic blood pressure of the pigs in the BWI-30 and BLWI-30 groups. (**B**, **C**) Lactic acid levels of the right mammary artery (**B**) and right external jugular vein (**C**) of the pigs in the BWI-30 and BLWI-30 groups. (**A–C**) Sham, no ischemia, $n = 3$; BWI-30, brain with 30-min warm ischemia, $n = 6$; BLWI-30, brain and liver with 30-min warm ischemia, $n = 7$. All replicates shown were biological replicates. I, ischemia; R, reperfusion. Mean ± SEM, 2-way ANOVA analysis; BLWI-30 versus BWI-30.

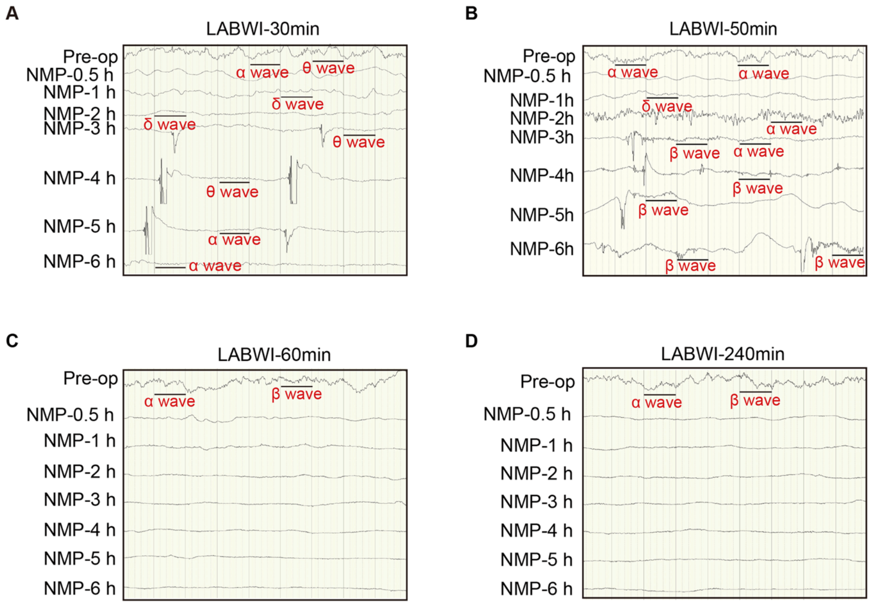

Figure EV2. Electrophysiological activity during ex vivo liver-assisted brain NMP.

(**A–D**) Electroencephalography results of a representative pig brain in the liver-assisted brain groups, where normothermic machine perfusion (NMP) followed intervals of 30-, 50-, 60-, or 240 min. LABWI, liver-assisted brain groups in which brain NMP was preceded by 30–240 min of warm ischemia time; Pre-op, Pre-operation.

 © The Author(s)

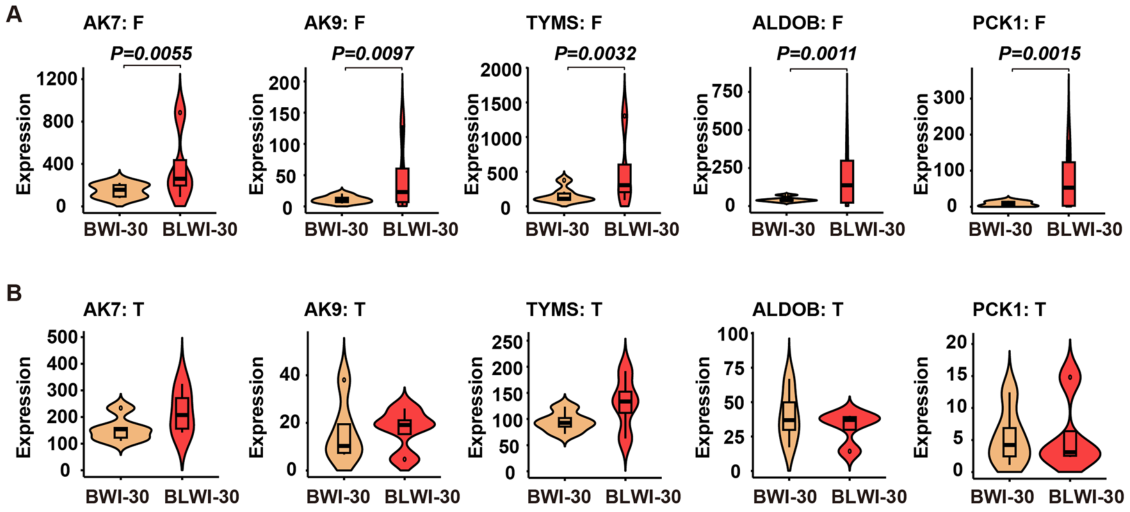

Figure EV3. Differentially expressed genes involved in metabolic processes in the global cerebral ischemia model.

(**A**, **B**) Violin plot illustrating the expression (reads per kilobase per million, RPKM) of differential genes in each of the biological replicates in the frontal lobe (BWI-30, $n = 6$; BLWI-30, $n = 4$) (**A**) or temporal lobe (BWI-30, $n = 5$; BLWI-30, $n = 4$) (**B**) of pigs. All replicates shown were biological replicates. (**A**, **B**) The vertical lines (whiskers) connecting the box represented the maximum and minimum values. The box signified the upper (75th percentiles) and lower quartiles (25th percentiles). The central band inside the box represents the median (50th percentiles). Outliers were shown. *P* values were calculated by the Wald test using the DESeq2 R package.

© The Author(s)

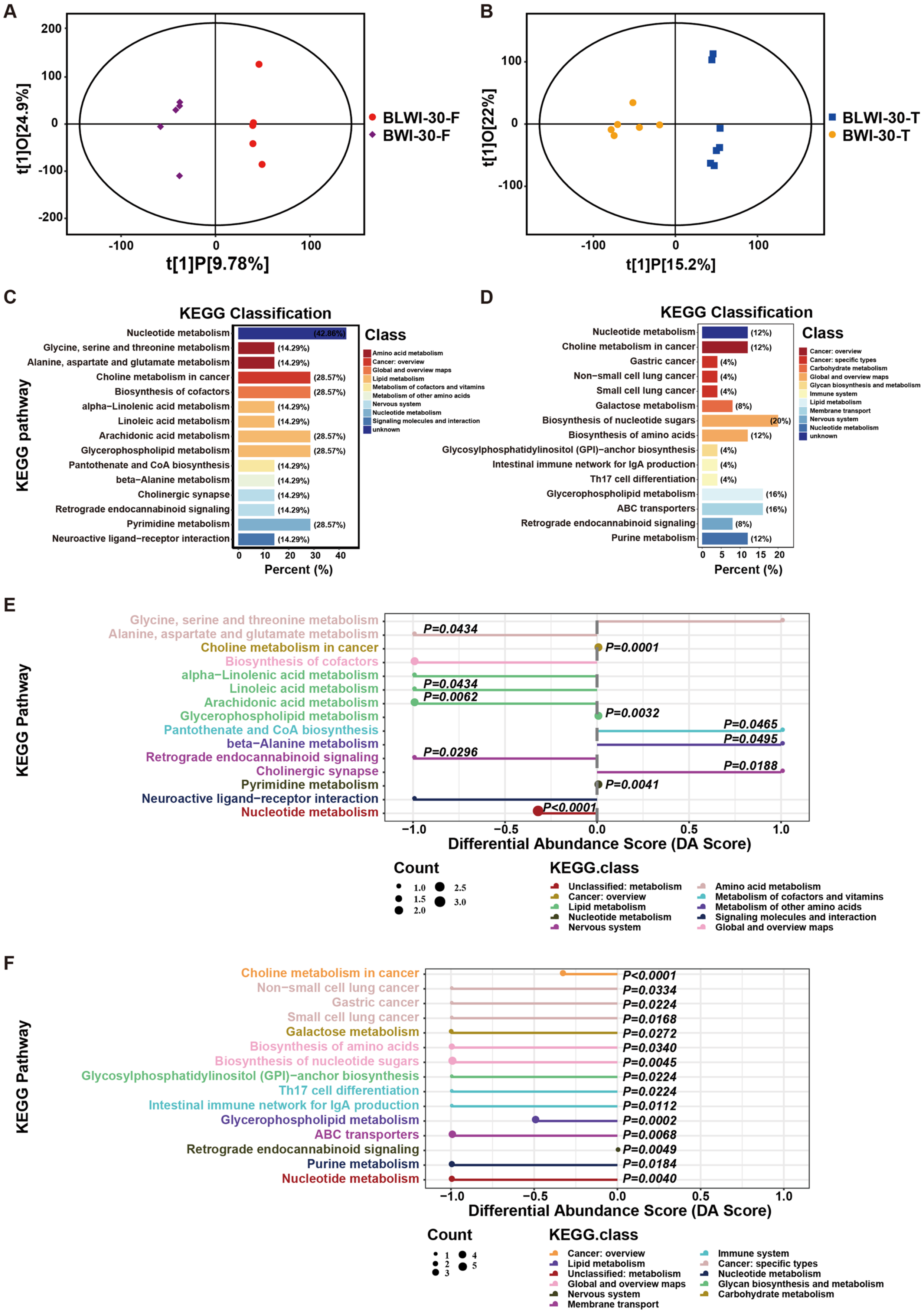
A
t[1]O[24.9%]
t[1]P[9.78%]
BLWI-30-F
BWI-30-F
B
t[1]O[22%]
t[1]P[15.2%]
BLWI-30-T
BWI-30-T
C
KEGG Classification
KEGG pathway
Nucleotide metabolism (42.86%)
Glycine, serine and threonine metabolism (14.29%)
Alanine, aspartate and glutamate metabolism (14.29%)
Choline metabolism in cancer (28.57%)
Biosynthesis of cofactors (28.57%)
alpha−Linolenic acid metabolism (14.29%)
Linoleic acid metabolism (14.29%)
Arachidonic acid metabolism (28.57%)
Glycerophospholipid metabolism (28.57%)
Pantothenate and CoA biosynthesis (14.29%)
beta−Alanine metabolism (14.29%)
Cholinergic synapse (14.29%)
Retrograde endocannabinoid signaling (14.29%)
Pyrimidine metabolism (28.57%)
Neuroactive ligand−receptor interaction (14.29%)
Percent (%)
Class
Amino acid metabolism
Cancer: overview
Global and overview maps
Lipid metabolism
Metabolism of cofactors and vitamins
Metabolism of other amino acids
Nervous system
Nucleotide metabolism
Signaling molecules and interaction
unknown
D
KEGG Classification
Nucleotide metabolism (12%)
Choline metabolism in cancer (12%)
Gastric cancer (4%)
Non−small cell lung cancer (4%)
Small cell lung cancer (4%)
Galactose metabolism (8%)
Biosynthesis of nucleotide sugars (20%)
Biosynthesis of amino acids (12%)
Glycosylphosphatidylinositol (GPI)−anchor biosynthesis (4%)
Intestinal immune network for IgA production (4%)
Th17 cell differentiation (4%)
Glycerophospholipid metabolism (16%)
ABC transporters (16%)
Retrograde endocannabinoid signaling (8%)
Purine metabolism (12%)
Percent (%)
Class
Cancer: overview
Cancer: specific types
Carbohydrate metabolism
Global and overview maps
Glycan biosynthesis and metabolism
Immune system
Lipid metabolism
Membrane transport
Nervous system
Nucleotide metabolism
unknown
E
KEGG Pathway
Glycine, serine and threonine metabolism
Alanine, aspartate and glutamate metabolism P=0.0434
Choline metabolism in cancer P=0.0001
Biosynthesis of cofactors
alpha−Linolenic acid metabolism
Linoleic acid metabolism P=0.0434
Arachidonic acid metabolism P=0.0062
Glycerophospholipid metabolism P=0.0032
Pantothenate and CoA biosynthesis P=0.0465
beta−Alanine metabolism P=0.0495
Retrograde endocannabinoid signaling P=0.0296
Cholinergic synapse P=0.0188
Pyrimidine metabolism P=0.0041
Neuroactive ligand−receptor interaction
Nucleotide metabolism P<0.0001
Differential Abundance Score (DA Score)
Count
KEGG.class
Unclassified: metabolism
Cancer: overview
Lipid metabolism
Nucleotide metabolism
Nervous system
Amino acid metabolism
Metabolism of cofactors and vitamins
Metabolism of other amino acids
Signaling molecules and interaction
Global and overview maps
F
KEGG Pathway
Choline metabolism in cancer P<0.0001
Non−small cell lung cancer P=0.0334
Gastric cancer P=0.0224
Small cell lung cancer P=0.0168
Galactose metabolism P=0.0272
Biosynthesis of amino acids P=0.0340
Biosynthesis of nucleotide sugars P=0.0045
Glycosylphosphatidylinositol (GPI)−anchor biosynthesis P=0.0224
Th17 cell differentiation P=0.0224
Intestinal immune network for IgA production P=0.0112
Glycerophospholipid metabolism P=0.0002
ABC transporters P=0.0068
Retrograde endocannabinoid signaling P=0.0049
Purine metabolism P=0.0184
Nucleotide metabolism P=0.0040
Differential Abundance Score (DA Score)
Count
KEGG.class
Cancer: overview
Lipid metabolism
Unclassified: metabolism
Global and overview maps
Nervous system
Membrane transport
Immune system
Cancer: specific types
Nucleotide metabolism
Glycan biosynthesis and metabolism
Carbohydrate metabolism

© The Author(s)

Figure EV4. Metabolome differences in pig brain tissues with and without simultaneous hepatic ischemia.

(**A**) Scatter plot depicting orthogonal projections to latent structures-discriminant analysis (OPLS-DA) results for the frontal lobe comparing the BLWI-30 and BWI-30 groups. (**B**) Scatter plot depicting the OPLS-DA data for the temporal lobe comparing the BLWI-30 and BWI-30 groups. (**C**, **D**) The bar plot displays the KEGG annotation of detected metabolites for the frontal lobe (**C**) and temporal lobe (**D**). The x-axis represents the percentage of identified metabolites in each KEGG class. (**E**, **F**) Depiction of overall changes in differential metabolites within a specific pathway for the frontal lobe (**E**) and temporal lobe (**F**). The Differential Abundance Score (DA Score) was calculated as the ratio of the difference between the upregulated metabolite count and the downregulated metabolite count on a specific pathway to the total count of metabolites on that pathway. (**A–F**) Frontal lobe: BWI-30, $n = 5$, BLWI-30, $n = 5$; temporal lobe: BWI-30, $n = 6$, BLWI-30, $n = 7$. All replicates shown were biological replicates. *P* values were calculated by Fisher's exact test (**E**, **F**) using the ggplot2 R package.

© The Author(s)

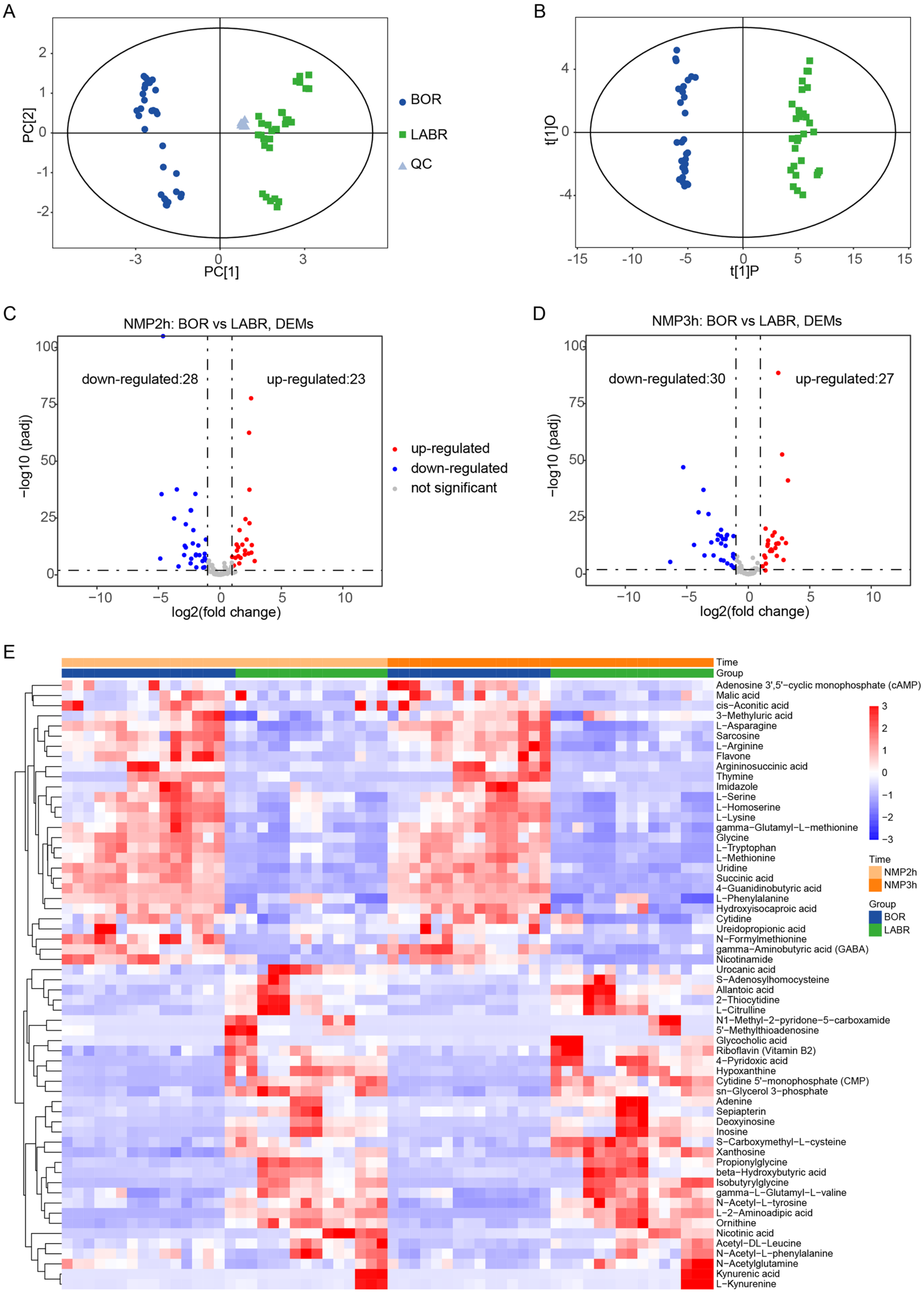
A
PC[2]
PC[1]
BOR
LABR
QC
B
t[1]O
t[1]P
C
NMP2h: BOR vs LABR, DEMs
down-regulated:28
up-regulated:23
-log10 (padj)
log2(fold change)
up-regulated
down-regulated
not significant
D
NMP3h: BOR vs LABR, DEMs
down-regulated:30
up-regulated:27
-log10 (padj)
log2(fold change)
E
Time
Group
Adenosine 3',5'-cyclic monophosphate (cAMP)
Malic acid
cis-Aconitic acid
3-Methyluric acid
L-Asparagine
Sarcosine
L-Arginine
Flavone
Argininosuccinic acid
Thymine
Imidazole
L-Serine
L-Homoserine
L-Lysine
gamma-Glutamyl-L-methionine
Glycine
L-Tryptophan
L-Methionine
Uridine
Succinic acid
4-Guanidinobutyric acid
L-Phenylalanine
Hydroxyisocaproic acid
Cytidine
Ureidopropionic acid
N-Formylmethionine
gamma-Aminobutyric acid (GABA)
Nicotinamide
Urocanic acid
S-Adenosylhomocysteine
Allantoic acid
2-Thiocytidine
L-Citrulline
N1-Methyl-2-pyridone-5-carboxamide
5'-Methylthioadenosine
Glycocholic acid
Riboflavin (Vitamin B2)
4-Pyridoxic acid
Hypoxanthine
Cytidine 5'-monophosphate (CMP)
sn-Glycerol 3-phosphate
Adenine
Sepiapterin
Deoxyinosine
Inosine
S-Carboxymethyl-L-cysteine
Xanthosine
Propionylglycine
beta-Hydroxybutyric acid
Isobutyrylglycine
gamma-L-Glutamyl-L-valine
N-Acetyl-L-tyrosine
L-2-Aminoadipic acid
Ornithine
Nicotinic acid
Acetyl-DL-Leucine
N-Acetyl-L-phenylalanine
N-Acetylglutamine
Kynurenic acid
L-Kynurenine
Time
NMP2h
NMP3h
Group
BOR
LABR

 © The Author(s)

Figure EV5. Metabolome differences in the perfusate serum during ex vivo pig brain NMP with and without a functioning liver support.

(**A**) Principal component analysis (PCA) showcasing the global variation within and between groups for the overall samples. (**B**) Scatter plot of orthogonal projections to latent structures-discriminant analysis (OPLS-DA) illustrating the differences between groups. (**C**, **D**) Volcano plot demonstrating the differential metabolites after 2 and 3 h of normothermic machine perfusion (NMP). (**E**) Heatmap displaying the z-score distribution of significantly different metabolites across various time points, with each row representing a metabolite. The heatmap was generated with the DESeq2 R package. (**A**–**E**) $n = 5$ pigs in each group, triplicate perfusate samples for technical replicates in each pig. (**C**, **D**) P values were calculated by the Wald test using the DESeq2 R package.

© The Author(s)
